# Chest Discomfort: Could Coronary Pathology Extend Beyond Atherosclerosis?

**DOI:** 10.3390/jcm15031185

**Published:** 2026-02-03

**Authors:** Ana Mladenovic Markovic, Ana Tomic, Miodrag Nisevic, Olga Nedeljkovic Arsenovic, Jelica Vukmirovic, Jelena Kostic, Aleksandar Filipovic, Ljiljana Bogdanovic, Vojislav Giga

**Affiliations:** 1 Centre for Radiology, University Clinical Centre of Serbia, 11000 Belgrade, Serbia; anatomic9977@gmail.com (A.T.); miodrag.nisevic@gmail.com (M.N.); olganedeljkovic@gmail.com (O.N.A.); jelica.doskovic@gmail.com (J.V.); jecko6yu@yahoo.com (J.K.); aleksandar.filipovic11@gmail.com (A.F.); 2 Faculty of Medicine, University of Belgrade, Dr Subotica Starijeg 8, 11000 Belgrade, Serbia; ljbogdanovic77@yahoo.com (L.B.); voja2011@yahoo.com (V.G.); 3 Institute for Pathology, University of Belgrade, 11000 Belgrade, Serbia; 4 Department of Cardiology, University Clinical Centre of Serbia, Faculty of Medicine, University of Belgrade, 11040 Belgrade, Serbia

**Keywords:** coronary arteries, MDCT, myocardial bridging, aneurysm, ectasia, fistula, dissection

## Abstract

**Background/Objectives**: Non-atherosclerotic pathological findings on coronary arteries involve various disorders that might lead to myocardial ischemia, independent of plaque complications and consequent lumen narrowing and obstruction. These patients often present with non-specific symptoms such as shortness of breath, rapid fatigue, and exertional chest tightness. When the underlying causes are non-atherosclerotic, these findings are frequently overlooked in radiology reports as a possible differential diagnosis. Therefore, the objective of this paper is to present the role of multidetector computed tomography (MD CT) coronary angiography in the diagnostic work-up of patients with rare but clinically valuable non-atherosclerotic pathological conditions of coronary arteries. **Methods**: We performed a literature search on Medline (via PubMed) for works presenting data on rare, non-occlusive, pathological findings on coronary arteries. **Results**: The review of the collected literature was performed in a narrative manner, intended to summarize mainly findings of imaging characteristics of non-occlusive pathologies: myocardial bridge, coronary aneurysm, ectasia, fistula, stenosis, and dissection. MD CT images of selected cases that were examined at our department, showing non-occlusive pathological changes in the coronary arteries, are displayed in planar and/or volume-rendered formats. **Conclusions**: Non-atherosclerotic abnormalities of the coronary vessel wall should be considered in the differential diagnosis of coronary causes of chest pain, dyspnea, and arrhythmias, as they may lead to both acute and chronic myocardial ischemia. Based on the presented literature and specific cases from our clinical practice, MD CT is shown to be an important tool for the rapid, non-invasive evaluation of non-atherosclerotic pathologies.

## 1. Introduction

Non-atherosclerotic pathological findings on coronary arteries involve various disorders that might lead to myocardial ischemia, independent of plaque complications and consequent lumen narrowing and obstruction. The most prevalent one is a deep myocardial bridge (MB), where a coronary artery (CA) dives in one segment deeper into the myocardium, 3 mm or more below its surface. Myocardial bridges—superficial or deep—are seen in about 40–80% of all autopsies, and even though mostly asymptomatic, deep MB can lead to episodes of myocardial ischemia due to systole compression of the bridging part of the CA. Hence, CA bridging frequently results in myocardial infarction with non-obstructive coronary arteries [1]. Coronary ectasia and aneurysm are also a rather unusual find, with their noted prevalence up to 4.9% for CA ectasias [2] and 5.3% for CA aneurysms [3]. Blood flow within the segmentally dilated CA can damage the endothelium, leading to thrombus formation within the lumen of the aneurysm, which can lead to distal CA embolization and obstruction [3]. Spontaneous coronary artery dissection and aortic dissection extending into the main coronary arteries are rare conditions that causes acute coronary syndrome (ACS) that requires emergency care and intervention, as its prevalence is recorded up to 4%, making it obliging for one of the differential causes of ACS [4,5]. Coronary arteriovenous fistulas are a congenital or acquired communications between coronary blood vessels and heart cavities, rather rare with a prevalence of 0.2% [6], but clinically important as they lead to chronic myocardial ischemia by shunting the blood away, through a coronary “steal” syndrome [7]. Coronary artery stenosis is an utmost rare anomaly when not attributed to the process of atherosclerosis, as it can be seen in certain cases with inflammatory, autoimmune vasculitis [8].

Multidetector computed tomography (MD CT) imaging offers benefits such as non-invasiveness, excellent sensitivity and specificity, and cost-effectiveness. Another benefit of MD CT coronary angiography lies in its capacity for 3D visualization of the ascending aorta, coronary tree, and adjacent tissues, which is crucial for surgical planning. It is crucial for evaluating the size and depth of myocardial bridges, accurately determining the presence of ectasia or aneurysm, as well as the potential existence of thrombus. Dependable visualization of wall thickening in vasculitis, arterial segments, and extensions is important. In addition, monitoring of fistulas and their connections with cardiac chambers and blood vessels, alongside accurate assessment of the fistula’s morphology and diameter is important. It is effective in evaluating intimal plaques and enables the measurement of hematoma attenuation in SCAD, along with the attenuation of adjacent structures. This procedure is rapid, non-invasive, easy to execute, utilizes minimal contrast agent, and enhances patient comfort due to a reduced chance of complications.

The objective of this paper is to present the non-occlusive pathological alterations of the CA that are considered to be a rare but clinically valuable find. We highlight these pathologies in cases when it comes to the differential diagnosis of coronary artery causes of chest pain, dyspnea, and arrhythmias that cannot always be associated with severe atherosclerosis. The role of MD CT coronary angiography as an imaging modality of choice in the detection of the aforementioned pathologies will be presented through various cases in our practice.

## 2. Literature Search

Using a narrative, non-systemic approach, we performed a search on Medline (via PubMed) for literature presenting data on rare, non-occlusive, pathological findings on coronary arteries. We conducted a keyword search in the literature for studies with titles or abstracts containing cases and data about aforementioned pathology, utilizing a combination of the following medical subject titles and keywords including: “myocardial bridge,” “coronary aneurism,” “coronary ectasia,” “coronary fistula,” “coronary stenosis,” “coronary dissection,” “SCAD,” “MDCT”, and “imaging”. No restrictions were imposed on the type of article. We have not indicated time limits, through September 2025. Titles and abstracts were initially evaluated, and possibly pertinent papers were discovered and assessed for inclusion or exclusion criteria. The inclusion criteria included studies that used MD CT to detect and present cases of aforementioned coronary non-occlusive, non-atherosclerotic pathologies. The exclusion criteria included articles that were not written in English, as well as articles that did not disclose diagnostic methods used to detect noted pathology, with scarce and inadequate reporting of coronary pathology characteristics and outcomes.

Identified items were assessed for relevance by the co-authors based on the noted objective and additional sources were identified by reviewing the references of articles retrieved in the literature search. Review of the collected literature was performed in a narrative manner, intended to summarize mainly findings of imaging characteristics of chosen pathologies. The noted sections were written in descriptive manner to gather and present essential information on the specified conditions. The thematic framework of this review was shaped by our clinical experience and the types of patients (and coronary pathologies) detected in our department, with illustrative, representative cases retrospectively assessed from our department’s repository of performed MD CT coronary angiographies, presented in accompanying figures.

## 3. Results and Discussion

### 3.1. Myocardial Bridge

Myocardial bridge (MB) is a condition when a section of the coronary blood vessel deviates from its normal path and continues deeper into the myocardium instead of emerging on the surface of the heart, leading to compression during systole. It is observed in a variety of frequencies of postmortem cases, ranging from 42% [9] with up to 78% [10], primarily in the central section of the left anterior descending artery (LAD) [11,12,13].

**Figure 1:**
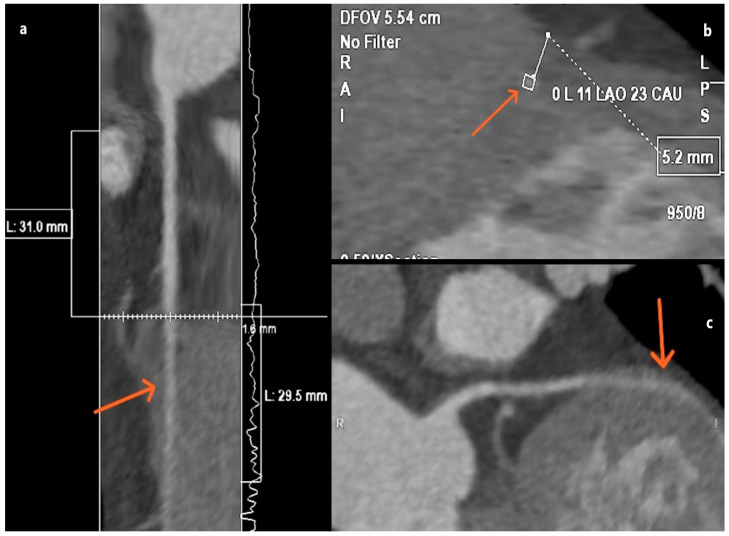
MD CT angiography plane images (**a**–**c**) showing a 5.2 mm (measurement in (**b**)) coronary myocardial bridge on LAD (arrow in (**a**–**c**)). Noted patient with a very deep myocardial bridge experienced pronounced anginal symptoms even with minimal exertion. The exercise stress echocardiogram was terminated within the first few minutes due to incidental ischemic findings. Management was predominantly conservative, consisting of lipid-lowering therapy with statins, along with beta-blockers and calcium channel blockers to reduce myocardial oxygen demand and alleviate symptoms. Due to the patient’s youth (early forties), invasive treatment (coronary artery bypass grafting CABG) was advised only if angina symptoms were to continue in non-exertional circumstances.

The literature distinguishes three forms of MB based on the depth of the bridging segment: superficial MB up to 2 mm [14], deep MB up to 5 mm, and very deep MB over 5 mm of overlying myocardium [15] (Figure 1). In addition to its depth and extent, the length of the myocardial bridge over 25 mm categorizes it as “long,” while the number of arteries impacted by the myocardial bridge is also significant [14,15].

The documented classifications are predicated on the circulatory effects of MB. The thickness and contractile properties of the overlying myocardium, in conjunction with recognized anatomical parameters like depth and length, directly affect the degree of systolic compression and its hemodynamic implications [15]. Namely, the portion of the coronary blood vessel immediately prior to the coronary bridge represents a predilection site for the formation of plaques, which often spread into the intramuscular part itself [16,17,18,19]. Plaque occurs as a result of increased pressure in the blood vessel wall, leading to damage to the endothelium [20,21]. Pathological studies show that people with a myocardial bridge deeper than 3 mm are more likely to develop an adverse coronary event [22].

The symptoms associated with myocardial bridges vary depending on whether the lesion is superficial or deep. A superficial myocardial bridge is often asymptomatic or presents with mild symptoms, such as chest discomfort. In contrast, patients with a deep myocardial bridge may experience angina-like manifestations even with minimal physical exertion or emotional stress, as well as shortness of breath, dizziness, palpitations, and even syncope [19,23]. It has been noted that myocardial infarctions that do not occur due to coronary occlusive disease, where patients exhibit normal coronary vascular appearance or a hemodynamically irrelevant lesion (less than 50%), sometimes result from a deep intramuscular bridge [1]. Given the relatively common epidemiological prevalence of these conditions, clinical suspicion of a MB should be suspected in all instances of typical or atypical chest pain, especially in young patients with a low likelihood of atherosclerosis who lack conventional cardiovascular risk factors [24].

MD CT coronary angiography is a highly precise type of scan that registers even the superficial MB, ones that go unnoticed and allows precise measurement of the length of the myocardial bridge, its depth, as well as insight regarding atherosclerosis distribution in segments close to MB [18,19,25,26]. It has several benefits compared to invasive and alternative non-invasive diagnostic techniques for evaluating the structural properties of MBs. Its efficacy is attributed to its superior spatial resolution, enhancing the detection rate of MBs relative to invasive coronary angiography, so enabling meticulous examination of the arterial wall and lumen, as well as the surrounding myocardium and neighboring structures, shown in three dimensions. This feature enables the examination of the artery’s path and classification of LAD-MBs according to their depth and length [27].

The management of symptomatic MB is problematic because to the absence of guideline-based management strategies. Medical therapy is regarded as the primary treatment, prioritizing beta-blockers as the preferred initial pharmacological choice, succeeded by calcium-channel blockers in patients with contraindications. Intensive clinical monitoring and proactive management of cardiovascular risk factors are crucial due to the correlation between MB and pre-MB segment atherosclerosis. In individuals exhibiting prolonged symptoms despite optimal medical treatment, revascularization may be contemplated; however, randomized evidence comparing medical therapy with percutaneous or surgical treatments are absent. Surgical interventions, such as coronary artery bypass grafting (CABG) or supra-arterial myotomy, sometimes referred to as “unroofing,” are efficacious but are reserved for refractory cases [14].

### 3.2. Pathological Dilatation of Coronary Arteries: Aneurysm and Ectasia

Coronary artery aneurysms (CAAs) are enlargements that exceed 1.5 times the usual diameter of the proximal portion of the artery. They are considered to be giant if their diameter transcends the reference vessel diameter by greater than four times or if they are above 8 mm in diameter [28]. CAAs have a reported incidence of 1.5% to 4.9% in adults [29].

**Figure 2:**
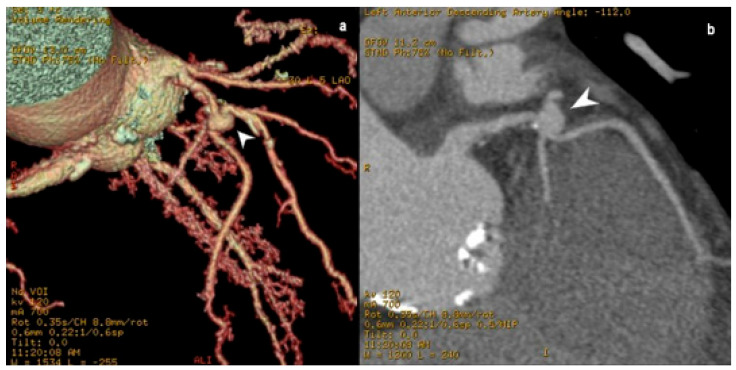
MD CT coronary angiography three-dimensional-volume rendered image (**a**) and planar view (**b**) images, showing a left anterior descending coronary artery saccular aneurysm (white arrowheads). The presented patient was managed conservatively, with radiological follow-up to monitor for aneurysmal enlargement, and was eventually referred to referent healthcare institution for surgical treatment.

**Figure 3:**
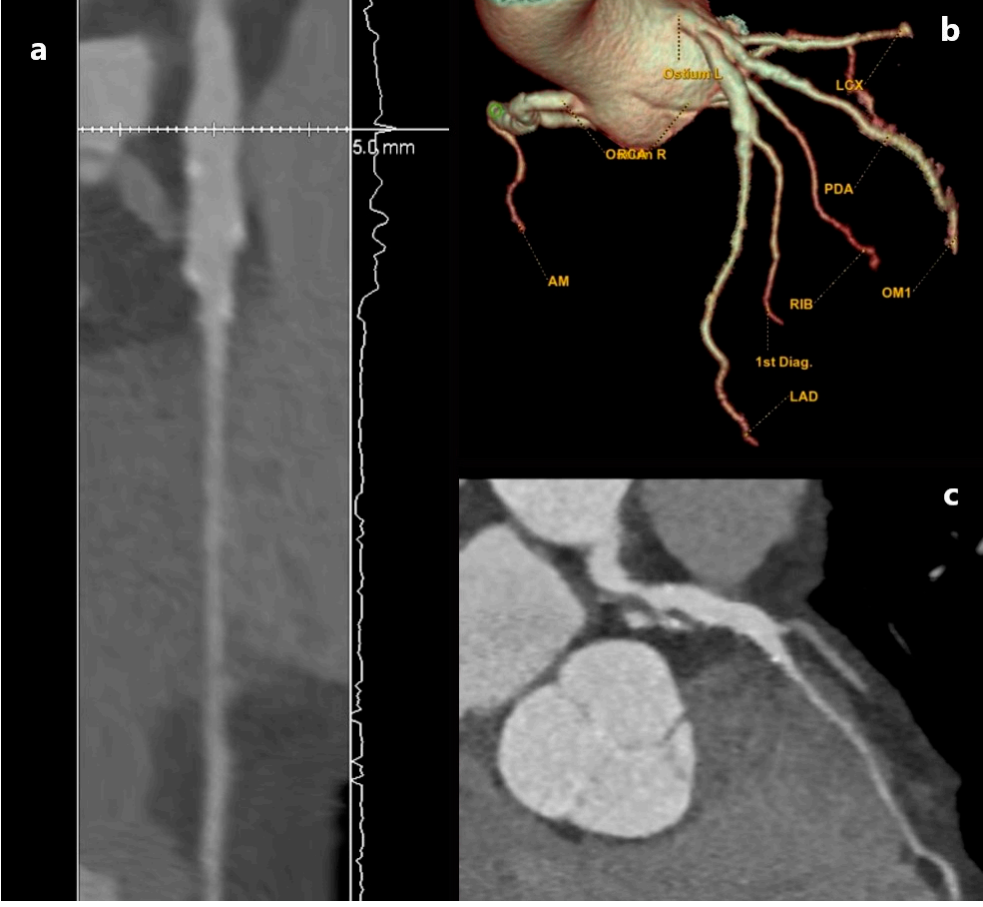
(**a**) MD CT coronary angiography angiographic (**b**) three-dimensional-volume rendered image and (**c**) planar view images, showing a left anterior descending coronary artery fusiform aneurysm. The presented patient underwent MDCT coronary angiography due to chest pain during exertion and shortness of breath. As all presented plaques caused less than 70% lumen reduction on examination, noted symptoms were due to either the presented aneurysms or concomitant myocardial bridges (seen in post-aneurysmal segment in (**b**)). Conservative treatment and radiological follow-up was indicated.

There are various ways to classify CAAs. The most commonly used classification system is in relation to the CAA diameter. In this context, they can be divided into saccular (Figure 2), in which the transverse diameter is larger than the longitudinal, and fusiform, in which the longitudinal diameter is larger than the transverse diameter [30] (Figure 3). In around 40–70% cases, CAA most often occurs on the right coronary artery (RCA), followed by the left circumflex artery (LCX) or the LAD, depending on the study [28]. Saccular aneurysms are most often localized on the LAD (Figure 2 and Figure 3). Depending on the integrity of the vessel wall, aneurysms can be classified as true or false aneurysms (pseudoaneurysms). Pseudoaneurysms are dilations of the vessel lumen, with a single or double layer of the vessel wall (instead of the normal three-layer structure), due to disruption of the tunica media and external elastic membrane. They are usually caused by blunt chest trauma or coronary intervention. In contrast, a true aneurysm involves all three layers of the blood vessel wall [31]. A less commonly used classification system is based on CAA etiology, classified into three groups: atherosclerotic, inflammatory, and non-inflammatory (related to congenital, inherited, and connective tissue disorders) [32]. The first two classes tend to be mentioned together in the literature [30], as atherosclerosis is the most common cause of CAAs in adults, with vessel wall inflammation considered the underlying mechanism [32].

**Figure 4:**
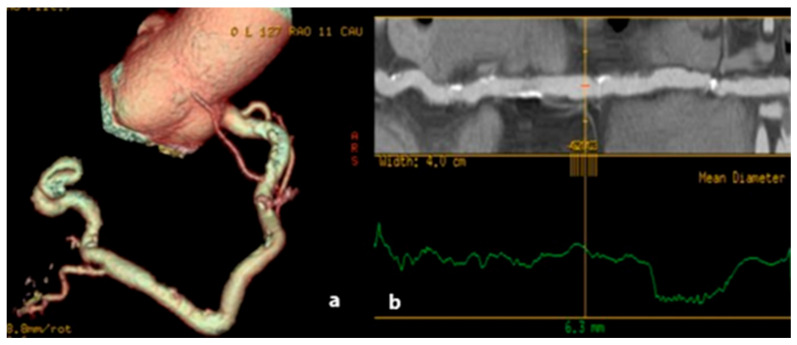
Three-dimensional-volume rendered image (**a**) and angiographic view (**b**) of type 3 coronary artery ectasia. The presented patient was managed conservatively, with radiological follow-up to monitor for further lumen enlargement.

**Figure 5:**
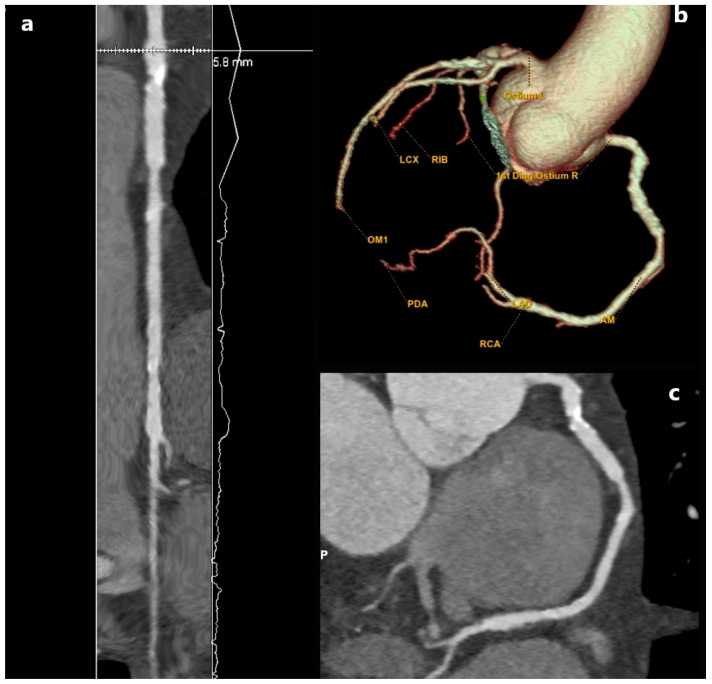
Three-dimensional-volume rendered image (**a**), angiographic view (**b**) and angiographic oblique view (**c**) of type 4 coronary artery ectasia. The presented patient underwent MDCT coronary angiography due to chest pain during exertion and shortness of breath. As all presented plaques caused less than 70% lumen reduction on examination, we could not state with certainty that symptoms were due to coronary artery disease (CAD) or the aneurysms themselves. Conservative treatment and radiological follow-up were indicated.

Ectasia is defined as local or generalized dilatation of the lumen of the coronary arteries and can be divided into 4 groups: type 1—diffuse dilatation of 2 or 3 blood vessels; type 2—diffuse dilatation of one blood vessel and localized disease of another blood vessel; type 3—diffuse ectasia of only one blood vessel and type 4—localized or segmental ectasia [2], as shown in Figure 4 and Figure 5. The exact mechanism of their development is unknown, but evidence points to a combination of genetic predisposition, common risk factors for coronary artery disease, and abnormal vessel wall metabolism [33].

Aneurysms and ectasias have been associated with diseases such as Takayashi arteritis, polyarteritis nodosa, Kawasaki disease (especially in children) [28], infectious diseases such as syphilis, trauma, and congenital anomalies [33]. Patients exhibit symptoms when slowed blood flow on the irregular inner surface of the aneurysm wall predisposes the formation of thrombus within the lumen of the aneurysm, which can result in distal embolization and myocardial infarction [34]. Aneurysm rupture that leads to cardiac tamponade can occur, especially in cases of giant CAA [35].

Associated obstructive atherosclerosis may result in dyspnea, stable angina pectoris or acute coronary syndrome [28]. However, the literature has shown that both types of aforementioned pathologies usually exist without any symptomatology and are incidentally detected during coronary angiography or CT angiography [31].

Aneurysms can be diagnosed through different imaging techniques that can evaluate the location and size of the aneurysm, the presence of blood clots within the blood vessel, the number and extent of the aneurysm, and the presence of any related complications, such as myocardial infarction [33]. Coronary angiography provides crucial data regarding the dimensions, morphology, position, and occurrence rate of the aneurysm, as well as the extent of atherosclerosis in the coronary arteries. Nevertheless, the utilization of non-invasive coronary angiography has been augmented due to the advent of MD CT. The introduction of MD CT has led to a higher occurrence of accidental discoveries of coronary aneurysms and ectasias. Three-dimensional reconstruction allows for detailed analysis of the dilatation in the coronary vessel, including its maximum diameter, shape, morphology, and any accompanying stenosis. It also provides information about the composition and location of the plaque in relation to the surrounding vasculature, as well as an analysis of the vessel lumen and wall composition. Additionally, it facilitates a clear understanding of the complex anatomical structures involved [31].

These disorders are frequently challenging to identify clinically. Patients may be asymptomatic or display symptoms of associated connective tissue disorders or vasculitis. The primary clinical manifestation is ischemic heart disease, characterized by exertional angina, with positive exercise stress tests observed even in the absence of significant atherosclerosis. This was associated with circulatory disturbances within the aneurysm, coupled with the formation of endoluminal thrombus resulting in embolization and microvascular impairment, ultimately culminating in the onset of acute coronary syndrome [30].

The optimal antithrombotic strategy for patients with CAA or CAE remains uncertain, with dual antiplatelet therapy or therapeutic anticoagulation generally reserved for symptomatic patients. PCI is required for aneurysmal or ectatic obstructed vessel in the setting of acute myocardial infarction. However, such interventions are associated with lower procedural success and higher rates of no-reflow and distal embolization [36]. Patients undergoing PCI of aneurysmal or ectatic CAs following ST-elevation myocardial infarction have been shown to experience increased intermediate-term mortality, as well as higher rates of stent thrombosis, target vessel revascularization, and recurrent myocardial infarction [37]. Compared with non-ectatic infarct-related arteries, ectatic vessels demonstrate a significantly higher thrombus burden [36] and more frequent use of adjunctive glycoprotein IIb/IIIa inhibitors and post-procedural anticoagulation [37]. Despite these challenges, selected cases may be successfully managed with PCI, including the use of overlapping stents to exclude large or pseudoaneurysms, achieving complete aneurysm sealing without compromise of side branches [38].

### 3.3. Coronary Arteriovenous Fistula

Coronary arteriovenous fistula (CAF) is a congenital or acquired communication between coronary blood vessels and heart cavities, CA themselves or with other blood vessels [39]. Majority of the CAFs are congenital and their incidence in the general population is estimated to be ranging from 0.1% [39] to 0.2% [40] on cardiac catheterization/invasive coronary angiography, while a coronary CT angiography reported an incidence of 0.9% [41].

**Figure 6:**
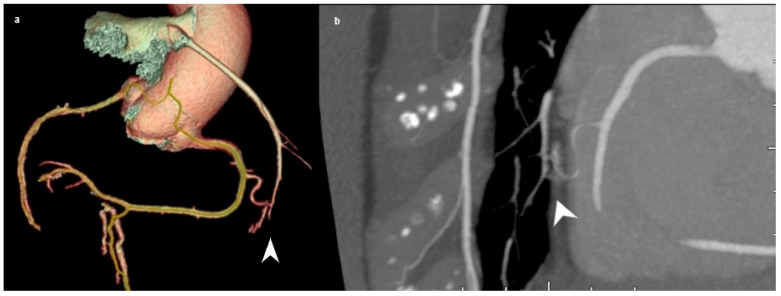
MD CT coronary angiography, three-dimensional-volume rendered image (**a**) and planar view image (**b**) showing RCA fistula with pulmonary artery branches (arrowheads) on angiographic view in Maximun Intensity Projection (MIP) mode. This fistula was clinically insignificant as this patient reported only a mild sensation of chest discomfort. Therefore, this was managed conservatively through clinical observation and regular follow-up.

**Figure 7:**
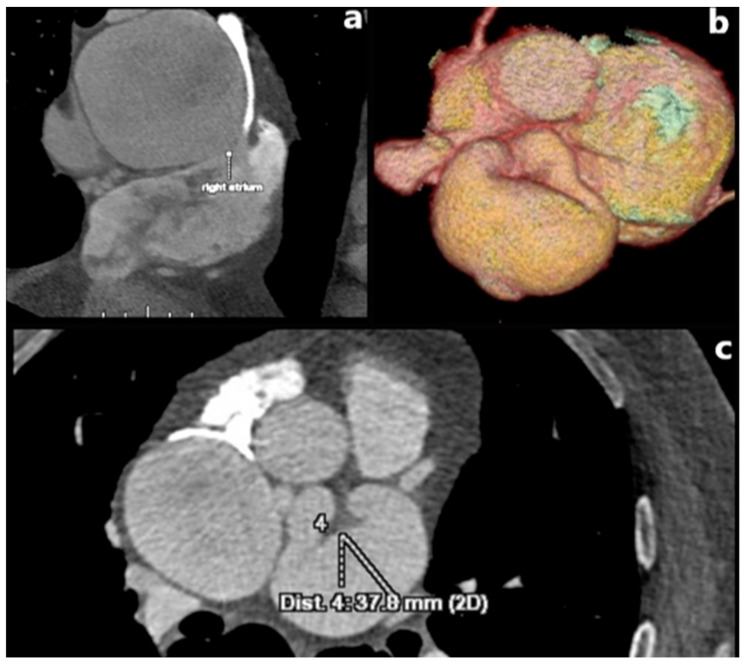
MD CT coronary angiography, (**a**) sagittal planar view (**b**) three-dimensional-volume rendered image and (**c**) axial planar view image, LAD fistula with right atrium (marked in text on (**a**)), with highly (aneurysmal) dilated LAD measuring up to 37.8 mm. This patient presented with pronounced anginal symptoms accompanied by occasional episodes of syncope, dyspnea, and electrocardiographic signs of myocardial ischemia. Surgical intervention was indicated to achieve definitive closure of the abnormal communication.

CAFs create a left-right shunt that in about 60% of cases occurs on the RCA [6], as shown in Figure 6. drainage of high-pressure blood in the arteries into a low-resistance venous system, chambers or coronary arteries depending on the size of the fistulous channel. The left main coronary artery (LMA), LAD, and LCX are less commonly affected (Figure 7), with rare cases reporting bilateral fistulas originating from both the RCA and LMA [42,43]. The most common connections with coronary blood vessels are those with the pulmonary artery or its branches [6,41,42,43], right ventricle or atrium of the heart, as well as the pulmonary veins [6] and coronary sinus [44]. CAF varies in size; they might be substantial, exceeding three times the diameter of a normal conduit (in practice, a fistula is deemed “huge” if its diameter surpasses 10 mm), or they may be little and enlarge with time.

The clinical manifestation of CAFs primarily hinges on the severity of the left-to-right shunt. Smaller CAFs are generally asymptomatic due to their minimal hemodynamic impact. As a result, the majority of adult patients are generally asymptomatic. However, larger shunts present clinically as fatigue, dyspnea, orthopnea, angina, arrhythmias, stroke, myocardial ischemia, or myocardial infarction [45], less often congestive heart failure, rupture and endocarditis [6,43]. Angina is the predominant symptom in cases where there is no coronary artery disease, and it arises when the fistulous aperture is enlarged due to the coronary steal phenomenon [46].

MD CT coronary angiography plays an important role in the evaluation of CAF by identifying their origin and termination, delineating their course, measuring vessel diameter, assessing aneurysmal dilatation, detecting thrombus, and demonstrating the jet phenomenon (a high-density, focal contrast jet is often seen at the site where the fistula enters the receiving chamber). Fistulae draining into the right heart chambers function as left-to-right shunts and may cause right ventricular volume overload. Termination in a low pressure space causes not only enlargement but also tortuosity of the fistulous coro-nary artery that leads to vascular wall degeneration, aneurysmatic dilatation and predis-position to rupture. Furthermore, the dilatation of the involved coronary artery may cause distortion of the aortic root and aortic valve disruption and regurgitation [47].

Management of the CAF depends upon its origin, pathway and size, presence of symptoms, as well as associated cardiovascular abnormalities. Available options for symptomatic fistula are surgical closure at the drainage site and transcatheterically aproach, using balloon catheters, covered stents or platinum microcoils [44], as well as vascular plugs used to perform percutaneous closure of the fistula [48]. In general, asymptomatic patients are monitored and do not require any treatment. In symptomatic patients, the choice of delivery catheter and embolization strategy for CAF closure depends on fistula size and anatomy. Small fistulas may be treated using microcatheters and pushable or detachable coils, with initial coil oversizing of approximately 30% to ensure adequate occlusion and additional coils deployed if residual flow persists [44]. Larger fistulas often require vascular occluder devices, such as the Amplatzer Vascular Plug, delivered via larger catheters, with device oversizing of approximately 50% and deployment at least 1 cm from the fistula origin to minimize the risk of prolapse or thrombus propagation [44]. There were documented instances of a vascular plug was used to perform percutaneous closure of the fistula, resulting in no residual flow and favorable hemodynamics at follow-up [48]. In complex or plexiform fistulas unsuitable for coil or occluder embolization, covered stent grafts may be used to exclude the lesion, although this approach necessitates prolonged antiplatelet therapy or anticoagulation due to an increased risk of stent thrombosis [44].

### 3.4. Coronary Artery Stenosis

Coronary artery stenosis is a rare congenital or acquired cardiac anomaly, frequently associated with hypoplasia of the artery ostium as well as the proximal segment of the artery [49]. Congenital hypoplasia of the coronary arteries presents as narrowing of the luminal diameter (less than 1.5 mm) in one or two of the three major coronary arteries, without compensatory branching, leading to symptoms of myocardial ischemia and potential sudden cardiac death [47].

**Figure 8:**
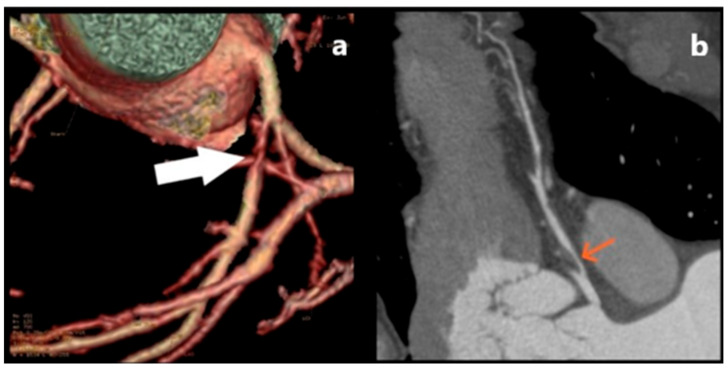
MD CT coronary angiography showing (**a**) three-dimensional-volume rendered image showing a segmental stenosis of the left anterior descending artery—LAD (white arrow)—as well as a (**b**) plane image of the stenosis (orange arrow). With noted high-grade stenosis of the proximal segment of the LAD secondary to vasculitis, the patient experienced severe anginal symptoms. In addition to optimal medical therapy, including beta-blockers, nitrates, and calcium channel blockers, surgical revascularization by coronary artery bypass graft (CABG) was indicated.

In cases of primary systemic vasculitis, main autoimmune disorder is characterized by necrotizing inflammation of blood vessels with subsequent fibrous changes in the vessel wall with stenosis of the vessel lumen (Figure 8). Immunoglobulin G4 (IgG4)-related disease can affect the cardiovascular system, including the coronary arteries. IgG4-related disease manifests in a variety of organs and tissues and is a well-known cause of aortitis with potential coronary involvement. This fibro-inflammatory condition has a tendency to form multiple tumefactive lesions causing stricture of the blood vessel [50,51]. This medical condition frequently manifests in diverse number of disorders, including giant cell arteritis, Takayasu’s arteritis, polyarteritis nodosa, Churg-Strauss syndrome, Wegener’s granulomatosis, Kawasaki disease and Henoch-Schönlein purpura [8]. This condition is potentially life-threatening, as it may present with myocardial ischemia and sudden death [49,52,53].

MD CT angiography is important for identifying these often-silent anomaly distinguishing non-occlusive coronary artery stenosis and lumen reduction caused by plaque. Noninvasive imaging in evaluation of IgG4-related cardiovascular disease (CVD) has an essential role in not only the diagnosis but also the management of this condition by defining anatomic landmarks and their relationships [54,55].

MD CT imaging is an effective modality for distinguishing inflammatory stenosis from atherosclerotic stenosis by analyzing plaque composition and wall features. Atherosclerosis manifests as luminal constriction caused by eccentric, frequently calcified plaques, together with the identification of a fatty component. In contrast, inflammatory stenosis, as shown in vasculitis, generally exhibits concentric, homogenous thickening of the artery wall with periarterial soft tissue thickening in acute phase of the disease [56], with circumferential fibrotic wall changes that lead to permanent stenosis in chronic phase. Additionally, novel methodologies for assessing concordant inflammation in these circumstances are being developed. A novel MD CT-derived imaging biomarker, the Pericoronary Fat Attenuation Index (pFAI), has demonstrated potential in this domain. The pFAI can identify phenotypic alterations and act as a marker for vascular inflammation by detecting gradients in pericoronary adipose tissue attenuation, potentially enhancing risk stratification in clinical practice, as pFAI has shown promise in predicting non-atherosclerotic coronary inflammation, including infective or immune-mediated vasculitis [57].

### 3.5. Coronary Artery Dissection

Coronary artery dissection can be either spontaneous or iatrogenic, i.e., after trauma. It represents an emergency condition with instant need for treatment. CA dissection occurs when a tear occurs in the wall of a blood vessel that can lead to slow flow, clot formation and occlusion, rhythm disturbances or sudden death. These occurrences are more frequent in women aged 40–50 who lack risk factors such as hypertension, hypercholesterolemia, or diabetes. The majority of coronary artery dissections occur on the LAD, but there have been cases of CA dissections on several blood vessels. Additionally, it is conceivable for dissections to occur repeatedly [4,5,58].

Spontaneous coronary artery dissection (SCAD) is a rare condition that is accompanied by severe chest pain that requires immediate intervention. Estimated prevalence of SCAD in patients with acute coronary syndrome (ACS) is between 1.7 and 4% [4,5]. However, SCAD typically presents as ACS, with reported cases of angina symptoms and registered acute ST segment elevation myocardial infarction (STEMI) [59]. It occurs more often in the population, and can even occur under 50 years of age. It has been shown that SCADs occur more often in younger women [4,60], as well as cases with pronounced tortuosity of coronary blood vessels, more often in people with fibromuscular dysplasia [61,62,63].

SCAD can be seen in three forms: SCAD Type 1 in which there is a dissection with the division of the lumen; Type 2 in which diffuse narrowing of the lumen of the blood vessel is seen at a length of 20 mm and more; Type 3 where tubular focal stenosis is seen at a length of less than 20 mm. In practice, Type 2 is most often encountered as it occurs in 67% of all SCAD cases [64].

SCAD is the predominant etiology of acute coronary syndrome in young women lacking traditional cardiovascular risk factors. The elevated occurrences of SCAD in young women, particularly those who are pregnant, postpartum, or taking oral contraceptives, indicates a potential influence of female sex hormones. Estrogen and progesterone may induce structural alterations in the artery wall, similar to other connective tissues, rendering it susceptible to rupture or dissection [65].

**Figure 9:**
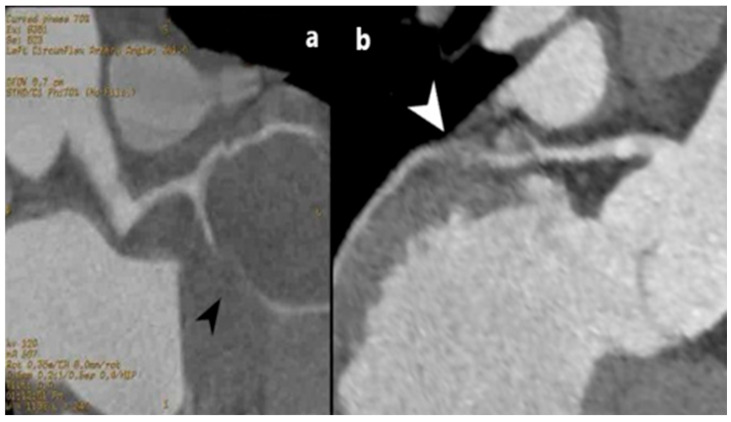
MD CT coronary angiography showing (**a**) spontaneous coronary artery dissection (black arrowhead); (**b**) plane image presenting the separation of the inner layer of a coronary artery wall, with formation of an intramural hematoma (white arrowhead). The presented patient was a postpartum woman that presented with symptoms of severe, radiating chest pain. She was initially referred for evaluation due to a clinical suspicion of pulmonary thromboembolism. She was managed conservatively using optimal medical therapy, which included dual antiplatelet agents and beta-blockers, in accordance with current clinical guidelines.

What can be detected by MD CT coronary angiography is the existence of two lumens, an intramural hematoma, a focal reaction of the surrounding fatty tissue often noted as “fat stranding” as well as the striking absence of CAD on other coronary arteries [66], as shown on Figure 9.

Differentiating SCAD, particularly when presenting as an intramural hematoma, from non-calcified soft-tissue plaque can be challenging on coronary CT angiography, especially in the absence of a visible intimal flap or documented prior coronary intervention. Both entities typically demonstrate low attenuation on CT. However, atherosclerotic plaques may show a heterogeneous internal composition, whereas SCAD is a homogeneous lesion with detectable intramural hematoma and perifocal fat-stranding. SCAD-related intra-mural hematomas are usually elongated, often extending over more than 20 mm, whereas non-calcified atherosclerotic plaques tend to be shorter, more focal. SCAD predominantly affects younger patients, more frequently women, while atherosclerotic plaques are more commonly observed in older patients with traditional cardiovascular risk factors. From an imaging perspective, SCAD typically involves angiographically normal or near-normal coronary arteries with minimal or absent atherosclerotic changes. In contrast, soft-tissue plaques are associated with evidence of atherosclerotic process of the vessel wall. SCAD is also frequently associated with marked coronary artery tortuosity and/or concomitant fibromuscular dysplasia. Conversely, positive arterial remodeling is a common feature of atherosclerotic plaque and is generally not observed in SCAD.

Another potential problem with MD CT diagnosis of SCAD involves distinguishing it from atherosclerotic plaque rupture. SCAD is a non-traumatic rupture of the coronary artery wall that forms a false lumen, impeding blood flow. It is distinct from atherosclerotic plaque rupture, as it often impacts younger, healthier vessels and is treated conservatively. Plaque rupture entails cholesterol-induced plaque breakdown and typically necessitates prompt intervention. MD CT angiography findings in SCAD, including the absence of atherosclerotic plaque, tapering luminal stenosis, abrupt luminal stenosis, luminal occlusion, intramural hematoma, dissection flap, and perivascular epicardial fat stranding. Affected segments frequently exhibit diffuse, extensive, or multi-vessel involvement. Plaque rupture generally exhibits localized, eccentric, or irregular stenosis accompanied by sudden occlusion, frequently linked to a “hazy” region indicative of thrombus, typically in segments containing pre-existing atherosclerotic plaque [67].

Conservative therapy is the optimal approach for stable patients with SCAD, as the majority of dissections resolve naturally. The initial treatment typically resembles that of acute coronary syndrome, encompassing dual antiplatelet medication, beta-blockers, and anticoagulation. However, potent antithrombotic drugs may theoretically impede healing of the intramural hematoma or exacerbate dissection, and thrombolysis should be avoided. Revascularization is designated for high-risk scenarios, including left main artery involvement, persistent ischemia, hemodynamic instability, sustained ventricular arrhythmias, or total arterial obstruction. When intervention is necessary, a cautious PCI strategy is advised, generally focusing on proximal segments to occlude the dissection entry, whereas distal dissections with maintained flow are generally handled conservatively. Coronary artery bypass grafting is indicated for left main dissections or when percutaneous coronary intervention is ineffective or impractical [68].

#### Aortic Dissection with Extension into the Coronary Arteries

**Figure 10:**
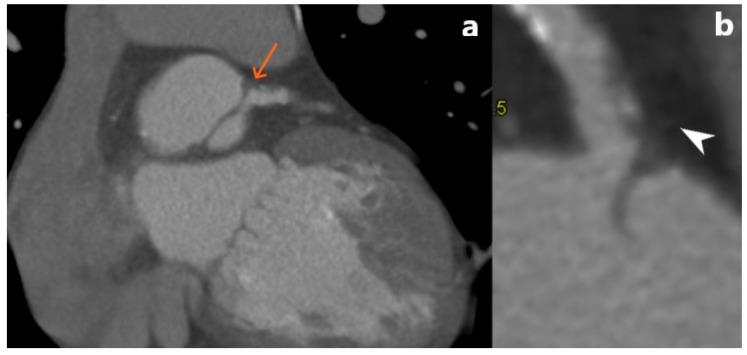
(**a**) Aortic root dissection spreading into the proximal portion of the LMA (arrow); (**b**) enlarged MD CT plane image showing the extension of the aortic dissection intimal flap within the proximal segment of the LMA (arrowhead). With noted pathologies on the aortic root as well as the LMA, the patient was referred to a vascular surgeon that indicated surgical treatment with elevated perioperative risk. However, the patient was not motivated, and therefore a conservative treatment with frequent follow-up was implemented.

Aortic dissections can extend into the main coronary arteries due to retrograde extension of the dissection flap [69] (Figure 10), than can sometimes it can be consequence of percutaneous coronary interventions [70]. Additionally, a significant risk factor for cardiovascular disorders such as hypertension, atherosclerosis, and arteriopathy in older adults is arterial stiffness. The key tissue conditions that contribute to arterial stiffening and endothelial dysfunction include the breakdown of elastic fibers and the elastic laminae as well as vascular inflammatory remodeling processes like fibro-calcification. This results in weakened, fragmented fibers that are unable to withstand elevated blood pressure, causing dissection tears [71].

The right coronary artery was often more implicated than the left. Aortic dissection involving LMA is extremely rare and has a high mortality rate [72]. In case of Stanford A (proximal aortic dissection, involving the aortic root or ascending aorta), myocardial infarction with acute STEMI can occur due to hypoperfusion of the coronary artery. This condition is potentially fatal due to the obstruction of the coronary ostia by the hematoma or intimal flap. On MD CT, a dissecting flap is seen in the aorta extending in coronary arteries, sometimes intramural hematoma, with delayed or absent flow in the vessel [73]. These dissections extending into the coronary arteries in a short segment are best visualized by MD CT coronarography.

### 3.6. MD CT—Technical Considerations

Current guidelines position MD CT as a first-line diagnostic modality for patients with low-to-intermediate risk of coronary artery disease, offering a cost-effective alternative to invasive coronary angiography. It can reliably exclude obstructive coronary disease and identify non-obstructive plaques that warrant preventive therapy, while reducing unnecessary invasive procedures [74,75].

To achieve these diagnostic outcomes, strict adherence to technical protocols should be ensured. A minimum of 64-slice MD CT is required, utilizing beta-blockers to achieve heart rates < 60 bpm. Crucially, modern protocols should adhere to ALARA (as low as reasonably achievable) principles, utilizing prospective ECG-triggering (“step-and-shoot”) and iterative reconstruction to reduce effective radiation doses from historical highs (>15 mSv) to current standards of 1–6 mSv [76,77].

Despite these advances, MD CT still has some limitations, particularly in patients with severe coronary calcification where “blooming artifacts” can cause the radiopaque calcium to appear larger than reality, obscuring the lumen and leading to false-positive estimations of stenosis [78]. Consequently, invasive coronary angiography remains the gold standard for patients with high pre-test probability, known severe calcification, or acute coronary syndromes where immediate therapeutic intervention is likely required.

## 4. Conclusions

Non-occlusive coronary artery pathology often presents with non-specific symptoms or may even remain clinically silent, yet it can underlie serious cardiovascular events and adverse outcomes. Therefore, non-atherosclerotic abnormalities of the coronary vessel wall should be considered in the differential diagnosis of coronary causes of chest pain, dyspnea, and arrhythmias, as they may lead to both acute and chronic myocardial ischemia. Based on the presented literature, our clinical practice and the representative cases, MD CT proves to be an important tool for the rapid, non-invasive evaluation of non-atherosclerotic pathologies. It provides highly accurate assessments of lesion size, morphology, vessel wall structure, and the anatomical relationships between these lesions and surrounding structures.

Although this type of examination or its interpretation largely depends on the patient’s heart rhythm and the skill of the radiologist, it is a fast and reliable method with additional analyses of cardiac enzymes, ECG, and a cardiologist’s examination to assess the patient’s condition, especially in emergency situations.

## Data Availability

The data presented in this study are available on request from the corresponding author due to the confidentiality of patient data, which is maintained to protect participants’ privacy and comply with ethical requirements.

## Abbreviations

The following abbreviations are used in this manuscript:

MD CT	multidetector computed tomography
CA	coronary artery
LAD	left anterior descending artery
LCX	left circumflex artery
LMA	left main artery
RCA	right coronary artery
MB	myocardial bridging
CAD	coronary artery disease
CAA	coronary artery aneurysm
CAE	coronary artery ectasia
CAF	coronary artery fistula
ACS	acute coronary syndrome
MIP	Maximum Intensity Projection
SCAD	spontaneous coronary artery dissection
STEMI	ST elevation myocardial infarction
